# Usefulness of circulating E-selectin to early detection of the atherosclerotic process in the Brazilian Longitudinal Study of Adult Health (ELSA-Brasil)

**DOI:** 10.1186/s13098-016-0133-9

**Published:** 2016-03-03

**Authors:** Bianca de Almeida-Pititto, Fernando Flexa Ribeiro-Filho, Marcio Sommer Bittencourt, Paulo A. Lotufo, Isabela Bensenor, Sandra R. G. Ferreira

**Affiliations:** School of Public Health, University of São Paulo, Av. Dr Arnaldo, 715, São Paulo, SP CEP: 01246-904 Brazil; Department of Internal Medicine, Federal University of Pará, Rua Augusto Corrêa, 01-Guamá, Belém, PA CEP: 66075-110 Brazil; Department of Internal Medicine, Centro de Pesquisa do Hospital Universitário, University of São Paulo, Av. Lineu Prestes 2565, 4th floor, São Paulo, SP CEP: 05508-000 Brazil

**Keywords:** E-selectin, Insulin resistance, Atherosclerosis, Endothelial dysfunction, Cardiovascular risk factors

## Abstract

**Background:**

This cross-sectional analysis evaluated whether determination of E-selectin concentrations could identify deterioration of cardiometabolic risk profile or subclinical atherosclerosis in individuals at low-to-moderate risk included in The Brazilian Longitudinal Study of Adult Health—ELSA-Brasil.

**Methods:**

A sample of 984 individuals from ELSA-Brasil (35–54 years) without cardiovascular disease or diabetes was stratified according to E-selectin tertiles. Traditional risk factors, inflammatory markers and categories of coronary artery calcium (CAC) scores were evaluated across the tertiles by ANOVA or Chi-squared test. In linear regression models, associations of E-selectin levels with insulin resistance index, adjusted for age, sex and adiposity were tested.

**Results:**

The mean age of the participants was 45.8 (SD 4.9) years and 55 % were women. Mean values of age, anthropometric data, biochemical variables and inflammatory status increased across E-selectin tertiles. Also, a gradual deterioration of the cardiometabolic profile was reflected by increments in frequencies (95 % CI) of BMI ≥ 25 kg/m^2^ [53.7 % (48.5–58.8), 61.0 % (56.1–66.5) and 64.2 % (59.0–69.4), p = 0.019], hypertension [18.0 % (14.1–22.8), 19.8 % (15.4–24.6) and 24.8 % (20.4–29.9), p = 0.048], pre-diabetes [62.5 % (57.4–68.3), 63.1 % (58.4–69.6) and 73.8 % (68.8–78.3), p = 0.003] and hypertriglyceridemia [22.4 % (17.9–27.2), 27.3 % (22.5–32.8) and 33.4 % (28.3–38.5), p = 0.013]. Insulinemia and HOMA-IR were independently associated with E-selectin concentration. A greater proportion of individuals with CAC scores different from zero was found in the third tertile when compared with the first and second tertiles (16.1 versus 11 %, p = 0.04, respectively).

**Conclusions:**

Direct associations of E-selectin with traditional risk factors slightly above their normal ranges, components of the metabolic syndrome, insulin resistance and presence of CAC suggest that this biomarker may indicate an initial atherogenic process.

## Background

Metabolic disorders such as type 2 diabetes mellitus (T2DM) and cardiovascular diseases (CVD) are major causes of death worldwide [1]. Despite some decrease in mortality rates, attributed to the control of risk factors (smoking, physical inactivity, elevated blood pressure and lipids and glucose levels), CVD is still the leading cause of disability adjusted life years in many developed and developing countries [2, 3].

Traditional cardiovascular risk factors are commonly clustered and insulin resistance is a pathophysiological link [4]. Low-grade inflammation and endothelial dysfunction are also involved in the insulin resistance syndrome. Both abnormalities are present in atherosclerosis and precede the development of T2DM [5, 6]. Some soluble molecules may be detected in circulation before these outcomes, which could represent an opportunity to identify atherogenesis prior to abnormalities in the traditional cardiometabolic risk profile [7, 8]. An early step in atherogenesis is the leukocytes adhesion to endothelium, triggered by a number of molecules, expressed in endothelial cells, that can be measured in circulation [9, 10]. Among those, the selectin family was shown to participate in this process [11] and has been associated with CVD and T2DM [7, 11, 12], but with the cardiometabolic profile particularly among low-to-moderate risk individuals has been less investigated.

The Longitudinal Study of Adult Health—ELSA-Brasil is a multicenter cohort study, including 15,105 active or retired employees of 6 Brazilian universities, aged 35–74, which will permit innovative investigation of multiple exposures and outcomes, particularly T2DM and cardiovascular events [13, 14]. This study offers an opportunity to examine novel circulating biomarkers of inflammation and atherogenesis that might be useful to further improvement in risk level detection. A subset of ELSA-Brasil participants was selected to assess the association of E-selectin with traditional risk factors as well as with structural lesion of atherosclerosis. We hypothesized that increased concentrations of E-selectin could be associated with the gradual progression of cardiometabolic risk profile in a subset of individuals at low-to-moderate risk.

## Methods

This cross-sectional study was conducted in a subset of the participants of one ELSA-Brasil center, the University of São Paulo, in Sao Paulo city. First examinations of 5056 individuals of ELSA-Sao Paulo were carried out from 2008 to 2010. For the purpose of the present analysis, after excluding diabetes and cardiovascular disease, a sample of 1000 individuals, aged 35–54 years, was randomly selected from the participants of São Paulo [15, 16], keeping the same proportions of sexes within the age groups (35–44 and 45–54 years) found in the main study. A total of 984 individuals with E-selectin determination were enrolled in this analysis. The institutional ethics committee approved the study and written consent was obtained from all individuals.

Participants had an initial interview using standardized questionnaires and then scheduled for physical examination and laboratory tests at the University Hospital. Body weight and height were measured using calibrated electronic scales and a fixed rigid stadiometer, while individuals wore light clothing without shoes. Body mass index (BMI) was calculated as weight (kilograms) divided by squared height (meters). Waist circumference was measured with an inextensible tape according to the WHO technique. Blood pressure (BP) was taken three times after a 5-min rest in the sitting position. The mean of the second and third measurements were used in the analyses.

After overnight fasting, blood samples were taken for several determinations. They underwent a 2-h 75-g oral glucose tolerance test. American Diabetes Association criteria were used to define categories of glucose tolerance, considering pre-diabetic those with impaired fasting glycemia (fasting plasma glucose ≥100 and <126 mg/dL and 2-h plasma glucose <140 mg/dL) and/or impaired glucose tolerance (2-h plasma glucose ≥140 mg/dL and <200 mg/dL and fasting plasma glucose <126 mg/dL) (ADA). Metabolic syndrome was defined by the presence of any three of the following components, according to international consensus criteria [17]:

- waist circumference ≥94 cm for men and ≥80 cm for women;
- systolic BP ≥130 or diastolic BP ≥85 mmHg (or antihypertensive treatment);
- fasting plasma glucose ≥100 and <125 mg/dL in the absence of antidiabetic agents;
- triglyceride concentration ≥150 mg/dL (or specific treatment);
- HDL-cholesterol <40 mg/dL for men and <50 mg/dL for women (or specific treatment).

Samples were immediately centrifuged and analyzed. Plasma glucose was measured by the hexokinase method (ADVIA Chemistry; Siemens, Deerfield, Illinois, USA). Total cholesterol was assessed by cholesterol oxidase method, enzymatic colorimetric, HDL-cholesterol by homogeneous colorimetric, without precipitation, triglycerides by enzymatic colorimetric (ADVIA Chemistry; Siemens, Deerfield, Illinois, USA); the low-density lipoprotein cholesterol concentrations were calculated by the Friedewald equation. When triglyceride concentration was greater than 400 mg/dL, the high-density lipoprotein cholesterol level was directly measured [18]. Aliquots were frozen at −80 °C for further determinations of hormones, apolipoproteins, markers of inflammation and adhesion molecule.

Insulin was determined by enzyme-linked immunoenzymatic assay—ELISA (Siemens, Tarrytown, USA). Homeostasis model assessment (HOMA-IR) was used to evaluate insulin resistance [19]. ELISA kits were also used for the determination of high-sensitivity C-reactive protein by immunochemistry (Dade Behring, Siemens, Marburg, Germany) and E-selectin (Abnova Corp, Taipei, Taiwan). Interleukin-6 (IL-6), interleukin-10 (IL-10) and tumor necrosis factor α (TNF-α) were determined by the Bio-Plex^®^ Pro Human Cytokine multiplex assay panel (Biorad, São Paulo, SP, Brazil). Intra-assay coefficients of variation ranged from 1.8 to 7.2. Inter-assay coefficients varied from 0.9 to 9.1, except for E-selectin (14.4 %). Sub-clinical inflammatory status was assessed through the ratio of pro-inflammatory and anti-inflammatory biomarkers, being IL-6/IL-10 and TNF-α/IL-10 ratios [20].

Results of coronary artery calcium score scanning were available for 947 individuals. They underwent ECG-gated unenhanced computed tomography to calculate CT calcium score measured according to Agatston et al. [21]. Analysis of coronary artery calcium was performed on a post processing workstation using dedicated CT calcium score analysis software. CAC was calculated and expressed as Agatston scores for standard of reference CT calcium score. Coronary artery calcium (CAC) score was categorized based as CAC = 0, 1–10 (mild), 11–100 (moderate), >100 (severe) [22, 23].

### Statistical analysis

Data are expressed as means and standard deviations (SD) or as medians and interquartile intervals. Distributions of some variables were skewed and thus were log-transformed before analysis to achieve normality. Values in tables were back-transformed to return to the natural scale. Participants were categorized into tertiles of E-selectin concentration to test its association with traditional cardiovascular risk factors, insulin resistance, inflammatory markers and CAC Agatston score. Data were compared by ANOVA with Bonferroni correction to identify the significant differences, which were marked with symbols. Frequencies were compared by Chi squared test and 95 % confidence intervals (95 % CI) were shown. Linear regression models were performed to evaluate independent associations of E-selectin levels with indices of insulin resistance, adjusted for age, sex and adiposity. Sensitivity analyses, excluding participants who were current smokers, were conducted to assess influence on variables. As results did not change, data from all the participants were shown. All statistical analyses were performed using the Statistical Package for Social Sciences, v. 19.0 for Windows (SPSS Inc., Chicago, Illinois, USA). A *p* value <0.05 was considered significant.

## Results

The mean age of the 998 participants was 45.8 (SD 4.9) years and 55 % were women. Nineteen percent had BMI ≥30 kg/m^2^ and 66 % were pre-diabetic. The majority of the sample (59 %) had at least 1 or 2 risk factors and 20 % were classified as having metabolic syndrome.

Decreasing proportions of women were observed across E- selectin tertiles, while mean values of age, anthropometric and biochemical variables increased (Table 1). Although the CRP did not differ across E-selectin tertiles, inflammatory status (assessed by the ratios of pro- and anti-inflammatory biomarkers) changed significantly across these categories.

**Table 1:** Number (% in parenthesis) or mean values (SD) of clinical variables including circulating biomarkers of a subset of ELSA-Brasil participants, according to tertiles of E-selectin

	Tertile1 N = 328 (4.6–55.3 ng/mL)	Tertile 2 N = 328 (55.4–99.2 ng/mL)	Tertile 3 N = 328 (≥99.2 ng/mL)	p value^b^	p value^a^
Women, n (%)	196 (59.8)	180 (54.9)	170 (48.8)	0.01	0.005
Age (years)	45.3 (5.0)	46.0 (4.7)	45.8 (4.9)	0.616	0.743
Body mass index (kg/m^2^)	25.9 (3.9)	26.1 (4.1)	27.2 (4.3)	<0.001^c, d^	<0.001
Waist circumference (cm)	85.0 (10.9)	86.1 (11.4)	90.0 (11.5)	<0.001^c, d^	<0.001
Systolic blood pressure (mmHg)	115.4 (14.2)	115.8 (13.9)	119.4 (15.2)	<0.001^c, d^	<0.001
Diastolic blood pressure (mmHg)	73.8 (10.1)	74.2 (10.3)	77.3 (10.6)	<0.001^c, d^	<0.001
Fasting plasma glucose (mg/dL)	102.3 (13.3)	101.9 (8.2)	103.9 (8.0)	<0.026^d^	0.037
2-hour plasma glucose (mg/dL)	118.5 (25.7)	121.6 (25.2)	122.1 (27.3)	0.170	0.082
HbA1c % (mmol/mol)	5.2 [32]	5.1 [31]	5.4 (36)	<0.001^c, d, e^	0.011
Total cholesterol (mg/dL)	205.4 (36.2)	208.2 (36.1)	215.0 (37.2)	0.003^d^	0.001
LDL-cholesterol (mg/dL)	126.9 (30.4)	127.3 (31.7)	132.7 (33.5)	0.037	0.021
HDL-cholesterol (mg/dL)	55.7 (13.4)	56.0 (13.6)	54.0 (12.6)	0.130	0.170
Triglycerides (mg/dL)	115.1 (64.4)	126.6 (71.5)	142.8 (89.1)	<0.001^c, d^	<0.001
Fasting insulin^f^ (µUI/ml)	5.7 (4.4)	7.7 (5.6)	8.5 (6.9)	<0.001^d, f^	<0.001
HOMA-IR	1.4 (1.2)	2.0 (1.5)	2.2 (1.8)	<0.001^d, e^	<0.001
C-reactive protein^f^ (mg/L)	1.2 (0.6–2.3)	1.1 (0.6–2.7)	1.3 (0.7–3.0)	0.244	0.122
IL-6/IL-10	4.3 (3.0–5.8)	4.4 (3.2–5.8)	4.6 (3.7–5.8)	0.010^c^	0.003
TNF-α/IL-10	2.7 (3.0–4.9)	3.7 (3.1–4.5)	3.8 (3.3–4.7)	0.044^c^	0.012

Values are means (SD) or medians (interquartile intervals), except for number of women (%) and for HbA1c reported in % (mmol/mol). SD for HbA1c was 0.6, 0.5 and 0.6 % for tertiles 1, 2 and 3 respectively

*Il* interleukin, *TNF-α* tumor necrosis factor-alpha

^a^p for trend or linear by linear

^b^p value obtained by Chi squared test or ANOVA with Bonferroni correction

^c^Tertile 1 versus Tertile 3

^d^Tertile 2 versus Tertile 3

^e^Tertile 1 versus Tertile 2

^f^Log-transformed variables for analysis

**Fig. 1:**
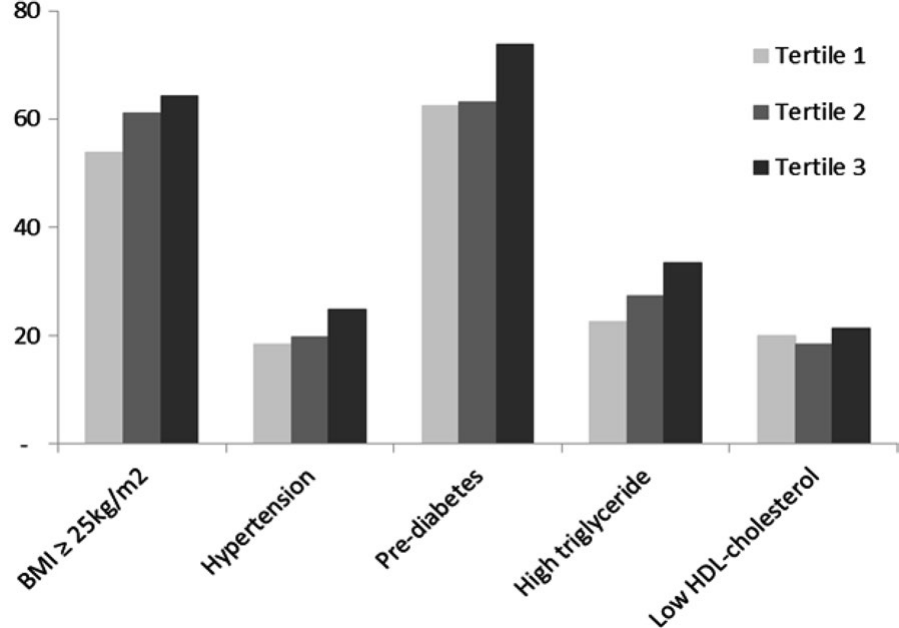
Frequencies (%) of traditional cardiovascular risk factors in a subset of ELSA-Brasil participants according to E-selectin tertiles. *BMI* body mass index (kg/m^2^). Pre-diabetes, impaired fasting glycemia (fasting plasma glucose ≥100 and <126 mg/dL and 2-h plasma glucose <140 mg/dL) and/or impaired glucose tolerance (2-h plasma glucose ≥140 and <200 mg/dL and fasting plasma glucose <126 mg/dL) (ADA). Hypertension, systolic BP ≥130 or diastolic BP ≥85 mmHg (or antihypertensive treatment). High triglyceride, triglyceride concentration ≥150 mg/dL (or specific treatment). Low HDL-cholesterol, HDL-cholesterol <40 mg/dL for men and <50 mg/dL for women (or specific treatment)

Figure 1 depicts the frequencies of traditional cardiovascular risk factors according to tertiles of E-selectin concentrations. The cardiometabolic profile showed a progressive deterioration across tertiles reflected by the differences in the mean frequencies (95 % CI) of BMI ≥25 kg/m^2^ [53.7 % (48.5–58.8), 61.0 % (56.1–66.5) and 64.2 % (59.0–69.4), p = 0.019], hypertension [18.0 % (14.1–22.8), 19.8 % (15.4–24.6) and 24.8 % (20.4–29.9), p 0.048], pre-diabetes [62.5 % (57.4–68.3), 63.1 % (58.4–69.6) and 73.8 % (68.8–78.3), p = 0.003] and hypertriglyceridemia [22.4 % (17.9–27.2), 27.3 % (22.5–32.8) and 33.4 % (28.3–38.5), p = 0.013], resulting in increasing proportions of individuals with the metabolic syndrome from the first to the second and third tertile of E-selectin [18.6 % (14.4–23.1), 19.5 % (15.0–24.2) and 28.3 % (23.6–33.4), p = 0.007, respectively].

In multiple linear regression analysis, the insulin resistance index HOMA-IR was independently associated with E-selectin concentrations, adjusted for sex and BMI (Table 2). When fasting insulin entered the model instead of HOMA-IR, the results did not change, showing its independent association with E-selectin. In both cases, the significant associations persisted when adjusted for waist circumference instead of BMI (data not shown).

**Table 2:** Final model of multiple linear regression for the association of E-selectin concentration (dependent variable) with insulin resistance index

	β	95 % CI	p value
Insulin resistance index	4.424	1.662–7.187	0.002
Sex (male)	10.347	3.054–17.639	0.005
Body mass index	1.200	0.188–2.213	0.020

Model adjusted for age (non-significant association)

*CI* confidence interval

Considering frequencies of CAC score categories, the third tertile of E-selectin levels had a greater proportion of individuals with CAC score different from zero or greater than 10 (Table 3). CAC score greater than 100 score was found in only 4.0 % (n = 12) of the participants of the third tertile and in 2.4 % (n = 14) of those included in first or second tertiles (p = 0.175).

## Discussion

The present study calls attention to the potential role of E-selectin concentration for earlier identification of the atherosclerotic process, since its circulating levels were shown to be associated with gradual deterioration of traditional risk factors (blood pressure, lipids and plasma glucose) even within near-normal ranges. Associations of E-selectin with components of the metabolic syndrome as well as with insulin resistance reinforce such statement. Despite the low frequency of structural coronary lesions in our sample, the presence of calcium in coronary arteries was also associated with higher E-selectin concentrations.

**Table 3:** Number of participants (%) in tertiles of E-selectin according to categories of coronary artery calcium (CAC) score

	Tertiles 1 + 2 N = 585	Tertile 3 N = 298	p value
CAC score > zero (%)	70 (11.4)	51 (16.1)	0.042
CAC score >10 (%)	46 (7.9)	36 (12.1)	0.041
CAC score >100 (%)	14 (2.4)	12 (4.0)	0.175

Chi square test was used

The rationale of examining adhesion molecule in middle-aged adults without T2DM or CVD took into consideration that novel circulating biomarkers of inflammation and atherogenesis might be useful to improve detection of risk level in further analyses [11, 24–27]. Although this study did not address cardiovascular risk stratification, clarifying the associations of these biomarkers in low-to-moderate risk condition could base future longitudinal analyses with such purpose.

Adhesion molecules, like selectins and CAMs, are stimulated by metabolic disturbances and their role for the development of the arterial plaque is recognized [10–12]. Although selectin has a major effect on endothelium activation, other tissues also express this molecule [10, 28]. At least in part, E-selectin is stimulated by inflammatory cytokines [29–31], which could explain the parallelism between TNF-α and E-selectin concentrations with the worsening of the metabolic profile observed in our study. Particularly, transcription of E-selectin occurs only on the endothelial cell surface, promoting leukocyte adhesion and migration to the subendothelial region, which triggers inflammatory response. In agreement with this underlying mechanism, in our study, inflammatory indexes (IL-6/IL-10 and TNF-α/IL-10 ratios), but not CRP, increased significantly across the tertiles of E-selectin. It is possible that low-grade inflammation detectable by CRP levels may be found in individuals at a higher cardiometabolic risk than those participants of the present study.

The progressive deterioration of traditional cardiovascular risk factors within near-normal values, in parallel with elevation of E-selectin concentration, could suggest the utility of this biomarker in identifying earlier abnormalities in cardiometabolic risk profile [10]. In previous cross-sectional study, our group reported a direct association of E-selectin and other inflammatory biomarkers with worsening of the glucose metabolism [32]. Although our study design precludes establishing cause-effect relationship, the observations of increasing frequencies of components of the metabolic syndrome across E-selectin tertiles are in agreement with this hypothesis. Actually, in a prospective study, endothelial dysfunction was shown to predict T2DM in women, independent of other risk factors [7].

Considering that insulin resistance is the central abnormality of the metabolic syndrome, we tested association of E-selectin with insulinemia and with HOMA-IR in multiple regression analyses. Associations of E-selectin with insulin resistance persisted after adjustment for sex and body adiposity. Along the same line, insulin sensitivity, as assessed by hyperinsulinemic euglycemic clamp, was found to be negatively correlated to E-selectin levels [8]. In addition, our data are in agreement with a study conducted in healthy subjects, in which adhesion molecules were associated with body mass and insulin resistance indexes [33].

Our findings were obtained in a large sample of middle-aged individuals whose body adiposity was near-normal, but their BMI and waist circumference gradually increased across E-selectin categories. There is some evidence that adipocyte-derived pro-inflammatory cytokines activate the production of endothelium proteins inducing insulin resistance [34]. A positive association of E-selectin with the insulin resistance index persisted even after adjusting for BMI. Therefore, we speculate that determination of E-selectin in blood could be useful to detect not only endothelium dysfunction but also a condition of insulin resistance independent of obesity.

Our observation of an association of HOMA-IR with E-selectin values reinforces a role of insulin resistance for atherosclerosis [5]. The present study had the opportunity of evaluating structural coronary lesions by computed tomography in a sub-sample of the ELSA-Brasil participants. CAC score has been recognized as an accurate method to detect coronary damage and predict events [21–23]. Due to the clinical characteristics of our participants, most had negative results for the presence of calcium in coronary arteries (CAC score equal to zero), which limited assessment of its trend across tertiles of E-selectin. Combining tertiles 1 and 2, we observed that the number of CAC scores different from zero was fewer than those in the third tertile. Considering the importance of E-selectin on the atherosclerotic process, our results may motivate further investigation of its potential as an earlier marker that could be a target for primary prevention. The cohort of ELSA-Brasil will permit not only being able to confirm these associations, but will provide the opportunity to investigate prospectively the impact of the exposure to this biomarker and others on several outcomes [13].

Our study has the strength of measuring this endothelium adhesion molecule in association with cardiometabolic risk factors and CAC score in a big sample size of middle-aged individuals at low-to-moderate risk. Other parameters of low-grade inflammation and insulin were also available. A limitation is related to the cross-sectional design that impedes inference of causality. A large inter-individual variability was observed similar to previously described.

## Conclusions

In summary, the findings of direct associations of E-selectin with traditional risk factors slightly above their normal ranges, with components of the metabolic syndrome, insulin resistance and CAC scores different from zero suggest that this biomarker could be indicating initial atherogenic process. The utility of E-selectin determination for the improvement of cardiovascular risk prediction deserves further investigation in longitudinal studies.

## Abbreviations

T2DM: type 2 diabetes mellitus
CVD: cardiovascular disease
IFG: impaired fasting glycemia
IGT: impaired glucose tolerance
CRP: C-reactive protein
BMI: body mass index
BP: blood pressure
IL-6: interleukin-6
IL-10: interleukin-10
CAC: coronary artery calcium
